# A 36-Class Bimodal ERP Brain-Computer Interface Using Location-Congruent Auditory-Tactile Stimuli

**DOI:** 10.3390/brainsci10080524

**Published:** 2020-08-06

**Authors:** Boyang Zhang, Zongtan Zhou, Jing Jiang

**Affiliations:** 1 College of Intelligence Science and Technology, National University of Defense Technology, Changsha 410073, China; zhangboyang09@nudt.edu.cn; 2 National Key Laboratory of Human Factors Engineering, China Astronaut Research and Training Center, Beijing 100094, China; jiangjingbuaa@buaa.edu.cn

**Keywords:** BCI, ERP, auditory, electro-tactile, bimodal stimulus, location-congruent, HRTF, BLDA, ANOVA, gaze-independent

## Abstract

To date, traditional visual-based event-related potential brain-computer interface (ERP-BCI) systems continue to dominate the mainstream BCI research. However, these conventional BCIs are unsuitable for the individuals who have partly or completely lost their vision. Considering the poor performance of gaze independent ERP-BCIs, it is necessary to study techniques to improve the performance of these BCI systems. In this paper, we developed a novel 36-class bimodal ERP-BCI system based on tactile and auditory stimuli, in which six-virtual-direction audio files produced via head related transfer functions (HRTF) were delivered through headphones and location-congruent electro-tactile stimuli were simultaneously delivered to the corresponding position using electrodes placed on the abdomen and waist. We selected the eight best channels, trained a Bayesian linear discriminant analysis (BLDA) classifier and acquired the optimal trial number for target selection in online process. The average online information transfer rate (ITR) of the bimodal ERP-BCI reached 11.66 bit/min, improvements of 35.11% and 36.69% compared to the auditory (8.63 bit/min) and tactile approaches (8.53 bit/min), respectively. The results demonstrate the performance of the bimodal system is superior to each unimodal system. These facts indicate that the proposed bimodal system has potential utility as a gaze-independent BCI in future real-world applications.

## 1. Introduction

Brain-computer interface (BCI) systems are novel human-computer interaction technology, which bypasses peripheral nerves and muscles and instead uses human brain activity to directly communicate with a computer or external devices [1,2,3]. BCI technology has developed rapidly since the 1970s [4,5,6]. In terms of signal acquisition methods, BCIs can be divided into two categories: invasive BCIs and non-invasive BCIs [7]. Although signals acquired using invasive BCIs end to be more accurate, scientific research is difficult to perform in general laboratory environments because of the need for brain surgery [8,9]. As such, non-invasive BCIs, most commonly using electroencephalography (EEG), have been widely applied in neural system and rehabilitation engineering [10,11]. In terms of EEG signal patterns, BCIs can be divided into spontaneous and evoked potential-based BCIs [12,13]. Specifically, event-related potential (ERP)-based BCIs are an important subset of evoked potential-based BCIs, in which, subjects are required to concentrate on infrequent target stimuli and ignore other non-target stimuli. Once the target event occurs, the EEG signals in the corresponding channels will change from a neutral ground state to an excited state [14]. Examples of ERPs include early components, such as the N200, and late components, such as the P300 and N400, etc. [15], the latency of which vary across individuals and ranges from 200 ms to 700 ms after target event onset [16].

Recently, researches on ERP-BCI have attracted extensive attention, and a number of practical application paradigms have been developed based on ERP-BCIs [17], which benefit from the following advantages: (1) target detection accuracy; (2) training time; (3) population adaptability; (4) information transfer rate (ITR); (5) paradigm flexibility, etc. [18,19]. Among the most of these researches, target events are usually based on visual stimuli, and require subjects to fix their eyesight on the stimulus interface at all times [20,21]. Nevertheless, for the individuals who have partly or completely lost their vision, traditional visual-based ERP-BCIs are ill-suited [22,23]. For instance, when a patient with paralysis manipulates an intelligent wheelchair with a visual-based ERP-BCI, he or she is required to fixate their attention onto a stimulus presentation screen causing them to ignore changes in their surroundings environment. To overcome these current limitations, auditory and tactile-based ERP-BCIs have emerged as a promising substitute for gaze-dependent systems [24]. Despite the usually lower ITR and classification accuracies, auditory- and tactile-based ERP-BCIs require few gaze shifts, and so visual feedback can be used to convey external scene instead of just being used as a simple stimulus source [25].

In respect to auditory-based ERP-BCIs, the first relevant paradigm was reported by Hill et al. in 2005, in which a binary target BCI was proposed by recognizing standard versus deviant voices [26]. Since 2010, Schreuder et al. realized a new auditory multi-class BCI paradigm and found that the performance of this auditory-based ERP-BCI improved by stimulating the subjects from different spatial orientations [27]. Guo et al. and Xu et al. further certified that active mental tasks increased target and non-target discrimination accuracy in an ERP-BCI paradigm, and thus improved the performance [28,29]. Building on these foundational researches, a number of studies have been expanded in the field in the last three years. Baykara et al. developed an auditory ERP-BCI system to spell words by using a 5 × 5 matrix of animal sounds and the corresponding ITR increased from 3.72 bit/min to 5.63 bit/min [30]. Moreover, Halder et al. optimized the aforementioned system and further improved the performance of the BCI when operated by subjects with motor impairments (i.e., the ITR to 5.78 bit/min, and accuracy to 92%) [31]. In addition, Miho et al. developed an ERP-BCI paradigm based on detection of six-direction virtual sound sources and found that the performance could be improved for both neurophysiological and behavioral responses, by means of shortening the stimulus onset asynchrony (SOA) [32].

More recently, tactile-based ERP-BCIs were initially explored by Brouwer et al. in 2010 [33]. In this paradigm, tactile stimuli were delivered via vibrating motors placed at different positions around the waist of the subject. Afterwards, moving towards the functional applications, Waal et al. developed a tactile ERP speller by classifying tactile stimuli delivered to different fingers and showed that a tactile speller provided a useful alternative to existing visual-based systems [34]. Subsequently, Kaufmann et al. designed another tactile ERP speller, in which the 26 letters in the English alphabet were divided into four groups and the tactile stimuli were delivered to four different areas of the subject’s left arm [35]. Furthermore, Kaufmann et al. also developed a wheelchair control system based on tactile ERP-BCI and investigated the use of a dynamic stopping method to improve the speed of the system [36]. Another wheelchair control system based on a tactile ERP-BCI was achieved by Herweg et al., in which elderly subjects participated in five sessions and tactors were placed on the legs, abdomen and back, and the system was validated on the elderly [37]. Most recently, Liu et al. proposed a tactile-based ERP-BCI paradigm for communication in 2018. In their study, the vibrators were positioned on the arm of the subjects to simulate a robotic arm. Subjects were able to successfully control a simulated mechanical arm with six degrees of freedom [38]. To date, vibration stimuli have been the most widely applied stimulus type in tactile-based ERP-BCI.

Different stimuli modalities are inherently useful as they can be used to evoke different potentials [39]. BCIs exist to leverage most of our five fundamental senses; however, the olfactory and gustatory perception channels are less developed from evolutionary standpoint, and it is difficult to reliably deliver stimuli. Researchers have already conducted studies on multisensory BCIs based on visual, auditory and tactile stimuli (though mainly visual-auditory and visual-tactile) [40,41]. Due to multisensory integration, the human brain synthesizes the neural information of each sensory channel independently, thus evoking a stronger ERP under multi-modal stimuli, leading to enhancement of the classification accuracy in ERP-BCIs [42,43,44,45]. For example, An et al. presented a visual-auditory ERPBCI speller for two different stimuli types (i.e., simultaneous redundant streams and interleaved independent streams), and explicitly explored the various combinations of these visual and auditory stimuli in a gaze independent BCI [46]. However, compared with each unimodal BCI, the performance of the bimodal BCI system did not reach a higher level. Recently, Sun et al. designed a novel improved visual-tactile bimodal stimulus ERP-BCI paradigm [47]. The average online ITR of the proposed pattern reached 12.49 bit/min, while the online ITR of the color-change pattern reached 8.87 bit/min on average. These results demonstrate that the picture-vibrate pattern achieved higher classification accuracy and online ITR than the color-change pattern. The limitations of the prior researches were mainly focused on three points: (1) the target for selection was too scarce; (2) using loudspeakers, the hardware load of the system increased significantly, which may give rise to mutual interference; (3) the time delay of the vibration tactile stimuli likely affects the instantaneous performance of the system. Consequently, our primary objective in this study is to overcome these three limitations and to further increase the ITR.

We proposed and developed a novel 36-class bimodal ERP-BCI system in this paper, in which the auditory and tactile stimuli were simultaneously generated from the same directions in a randomized order. Specifically, six-virtual-direction audio files were delivered from “1” to “6” through headphones, and the male voices were used for row selection as well as the female voices for column selection. In addition, electro-tactile stimuli were delivered by six pairs of electrodes placed around the abdomen and waist of the subjects corresponding with the angle of each virtual direction generated by the head related transfer functions (HRTF). Moreover, by offline data training, we selected the eight best channels, trained the Bayesian linear discriminant analysis (BLDA) classifier and obtained the optimal trial number and used these parameters in the online signal processing.

## 2. Materials and Methods

### 2.1. Subjects

There were 12 healthy subjects (age 18–25, mean age 20.83, denoted as S1–S12) in the experiments. All subjects had no history of neurological or psychological disorders. In addition, the subjects had normal hearing and normal touch sense in the waist and abdomen areas, and had no difficulties localizing sounds in space or history of trauma. None of them had participated in the experiments based on auditory or tactile BCI. Before the experiments, all the subjects were familiar with the purpose and tasks and signed the informed consent form. The study was approved by the ethical approval committee of Xiangya Hospital, Central South University, with the ethical approval code of “2018101045”, and the approval date was 10 October 2018.

### 2.2. The BCI Design

**Figure 1:**
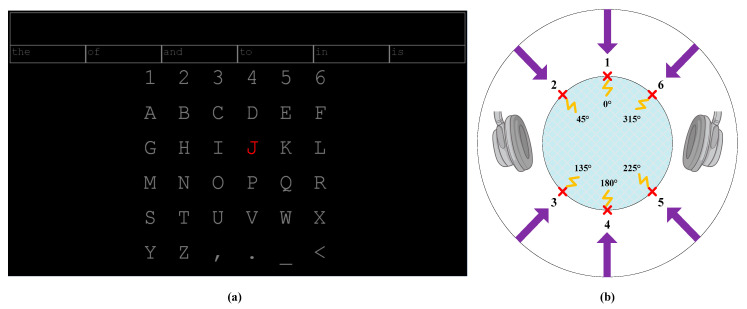
Illustration of the character distribution of the 36-class brain-computer interface (BCI) system and the bimodal stimulus paradigm. In subfigure (**a**), the prompted character “J”, as an example, is marked red. In subfigure (**b**), the stimuli were delivered from corresponding location. Each yellow broken line represents a pair of electrical tactile electrodes placed at waist level. The arrows represent the directions of auditory stimuli delivered from headphones. The numbers identify the stimulus codes (location-congruent bimodal stimuli from “1” to “6”).

In our study, the BCI systems were composed of two portions: (1) user interface and (2) bimodal stimulus presentation, based on the MATLAB (MathWorks, Inc., Natick, MA, USA) platform. The user interface was used to present the characters on a 23.6-LCD monitor with a 1920 × 1080 resolution and a 60 Hz refresh rate. The distance between the monitor and the subject’s eyes was set to 70 cm. The character presentation was controlled by PTB-3 in MATLAB [48]. As shown in Figure 1a, a total of 36 (6 × 6) characters (26 letters, six digits, three punctuations and an operator) were presented on the screen. Each character was presented within a 40 × 40-pixel square. The vertical and horizontal distances between two neighboring characters were both 50 pixels. We employed the classical standard BCI speller with a 6 × 6 matrix, in which the target character was selected by the row/column (RC) mode. In the RC mode, the selection of each item in the matrix was grouped into row and column. The bimodal stimuli were presented in a random order to select the row and column indexes of the target character in succession. The intersection of the row and column determined by the ERP detection algorithms was identified as the target character. For the individuals who have partly or completely lost their vision, if their auditory and tactile functions are normal, they can also use the BCI system by memorizing the coordinates of each character, instead of by vision information. For example, when a user wishes to spell “J”, they will perform the following steps:

- Recognize “J” is in the 3rd row. Focus on the stimulus “3” (at 135°) and count the number of randomly delivered repetitions of the desired stimulus;
- The computer determines the user is focusing on the third row after the preset number of repetitions of stimuli is reached;
- Recognize “J” is in the 4th column. Focus on the stimulus “4” (at 180°) and count the number of randomly delivered repetitions of the desired stimulus;
- Lastly, computer determines the user was focusing on “4”. Consequently, the character “J” is presented on the screen if both the row and column indexes are correctly selected.

The bimodal stimulus presentation portion was composed of two major components: a stereo headset for playing the directional audio files, and an electrical stimulation controller to deliver the electro-tactile stimulus. The stimulus duration was set to 260 ms, and the inter-stimulus interval was set to 250 ms. A trial was defined as one complete stimulus cycle, in which each of the six stimuli were delivered once. At least two cycles were used for the selection of the row and column index. The direction of the stimulus was coded from “1” to “6”. The commands for controlling the onset of the stimuli were simultaneously sent from MATLAB to the electrical stimulation controller and stereo headset, which ensured the time synchronization of the auditory and tactile stimuli. A time break (1 s) was given after each selection, during which time the next target character turned red to prompt subjects to the next selection task. In addition, there was a short pause (0.75 s) when switching between the selection of the row and column index, so that subjects could pay attention to the column index in which the target character was located. We developed auditory, tactile and bimodal BCI to compare the unimodal system with the bimodal system. Additional details of the stimulation mechanism are shown in Figure 1b.

### 2.3. The Stimulus Design

#### 2.3.1. Auditory Stimulus Design

The headphones delivered the auditory stimuli in six different directions (“1” at 0° relative to where the subjects faced, “2” at 45°, “3” at 135°, “4” at 180°, “5” at 225° and “6” at 315° in counterclockwise order) (see Figure 1b). To generate the sounds in six directions, we employed the HRTF [49] to turn two-channel audio files into the virtual six-direction audio files. HRTF is defined as:

(1) $$\{\begin{array}{l} HL=PL\left(r,\theta ,\varphi ,\omega ,\alpha \right)/PO\left(r,\omega \right)\\ HR=PR\left(r,\theta ,\varphi ,\omega ,\alpha \right)/PO\left(r,\omega \right) \end{array}$$

where *PL* and *PR* are the complex sound pressure generated by the simple harmonic point sound source in the left and right ears of the listener, respectively. *PO* is the plural sound pressure at the center of the head. *HL*, *HR* is the function of the horizontal azimuth angle of the sound source *θ*, the elevation angle of the sound source *φ*, the distance from the sound source to the center of the head *r*, the angular frequency of the sound wave *ω*, and the head size *α*.

In our approach, the six ordinary audio files used for stimuli were “1” to “6” in the Chinese language and each was of equal duration. Then, the audio files were transformed to a virtual direction using the HRTF. After transformation, each stimulus had duration of 260 ms. The generated audio files were divided into male and female voice files for the selection of the row index and the column index, respectively. The male voice was used to select row index and the female voice was used to select column index, which made it convenience for the subject to distinguish between row selection and column selection. Consequently, a total of 12 audio files were generated. The volume of the headphone was set to approximately 80 db. Headphones were employed instead of loudspeakers to reduce the system setup time and the effects of disruptive surrounding noise. Since active mental task has been proved to enhance the performance of auditory-based BCI, during the experiments, the subjects were asked to mentally count the number of repetitions of the desired target [28].

#### 2.3.2. Electro-Tactile Stimulus Design

The electro-tactile stimuli were delivered through a six-channel current controlled electrical stimulation generator (Neuracle Tech., Changzhou, China). Compared to mechanical approaches, such as vibration motors, electrical approaches have numerous advantages including easier adjustability of stimulus intensity and lower time delay. Six pairs of two standard self-adhesive disposable electrocardiogram (ECG) stimulation electrodes (Ch50rb, HealForce Bio-Meditech, Shanghai, China) were around the subject’s abdomen and waist with the same angle of each virtual direction of auditory stimulus. The electro-tactile stimuli were generated by the local current in each pair of electrodes on the skin surface. The electrodes of a given pair were 5 cm apart and interfaced to the computer. The instructions were delivered through a MATLAB program.

**Figure 2:**
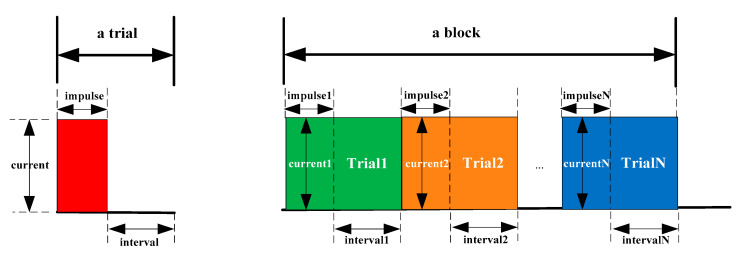
Illustration of electro-tactile impulse stimuli.

The generators provided electro-tactile stimuli by means of discrete electrical impulses. The current intensity ranged from 0.1 to 2 mA, the impulse width ranged from 0.1 to 6553.5 ms, and the time interval ranges from 10 to 65,535 ms. These types of generators are safe, portable and robust. Before placing the electrodes at corresponding locations on the subject’s waist, the candidate electrode locations were wiped with exfoliating cream twice to reduce the skin impedance so that subjects could more easily to perceive the electro-tactile stimuli. For the safety and comfort of the subjects, we set the current intensity (<2 mA) in each direction separately. Moreover, the impulse width was set to 260 ms (the same duration of each audio file), while the time interval was fixed to 250 ms. The mechanic of electro-tactile stimuli is shown in Figure 2.

#### 2.3.3. Bimodal Stimulus Design

In the bimodal stimulus paradigm, ERPs were evoked via location-congruent auditory and electro-tactile stimuli simultaneously. As shown in Figure 1b, the bimodal stimuli were delivered from six directions. Furthermore, we delivered the virtual direction auditory and electro-tactile stimuli at corresponding positions. This is a location-congruent paradigm, and the coded number “1” to “6” represents the directional information of the bimodal stimulus system. When prompted in a certain direction, the subjects need to focus on the auditory and tactile stimuli in that direction, while ignoring the stimuli from the other directions.

### 2.4. Experiment Procedure

In our approach, the experiments were conducted in a quiet laboratory. The subjects were instructed to sit on the chair and face the LCD monitor place in front of them. The subjects were asked kept their eyes focused on the red prompted character so that blinking and eye movements were reduced. The subjects performed an exercise to familiarize themselves with the experimental tasks before each session. Each subject performed auditory, tactile and bimodal paradigm in three different sessions. We set the sessions in a random order to avert the sequence effect.

**Figure 3:**
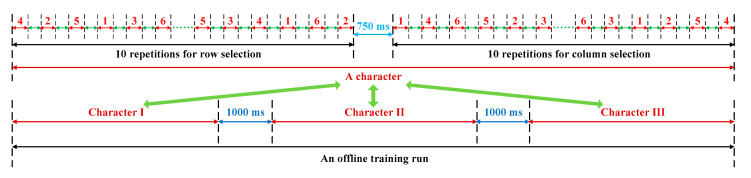
Stimulus sequence generation.

In each session, the subjects conducted 15 runs. As shown in Figure 3, each run was comprised of three character selections. Each character selection consisted of 10 row and column repetitions for each stimulus in a randomized order. In each repetition, the subjects were asked to pay attention to one number at a time for each row/column selection. An example sequence might be “4-2-5-1-3-6”. To avert prejudgment, the order of these coded numbers was randomized. The ERP-BCI system detected the row and column numbers focused by subjects. The intersection of the row and column was identified as the target character. The first ten runs were in the offline phase, which was used for channel selection, the ERP classifier training and the optimal trial selection; in the online phase, we applied the next five runs. Based on the offline and online phase, we compared the performance between bimodal and unimodal BCI. Between two adjacent runs, we arranged a five-minute interval for each subject to prevent fatigue.

## 3. Signal Processing

Offline training procedure:

- Signal acquisition and preprocessing;
- Channel selection;
- BLDA training;
- Trial number optimization.

Online selection procedure:

- Signal acquisition and preprocessing;
- ERP feature extraction;
- Target selection.

More details on the signal processing are as below.

### 3.1. Signal Acquisition and Preprocessing

**Figure 4:**
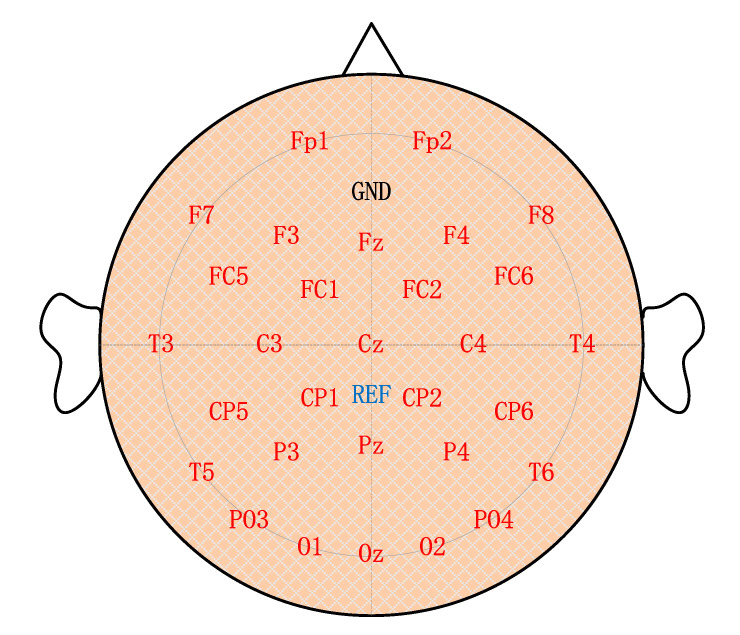
Configuration of electrode locations.

The raw EEG signals were acquired utilizing a standard EEG cap (Neuracle Tech., Changzhou, China) with 32 active Ag/AgCl electrodes following the international 10−20 system. The signals were sampled at a sampling rate of 250 Hz and amplified with a wireless amplifier (Neuracle, Changzhou, China). The configuration of electrode locations was in Figure 4. The impedance of each electrode was kept below 10 kΩ.

Both offline and online, signal preprocessing was the required procedure. Initially, the raw EEG data were treated with a 50 Hz notch filter and then with a 0.05−45 Hz band-pass filter. Then, the 1000 ms segment after each stimulus onset was extracted for the ERP feature analysis. In addition, the 400 ms pre-stimulus portion was subtracted from the post-stimulus segment to estimate a mean baseline amplitude. Finally, the data segments were down sampled from 250 to 25 Hz to acquire the major ERP information by an anti-aliasing moving average filter.

### 3.2. Channel Selection

In practical applications, the number of EEG channels used in the classification should be as small as possible to reduce the computational complexity and hardware requirements. However, it is inherently difficult to select the smallest channel set while maximizing detection accuracy because EEG feature spaces vary across individuals. The bilateral temporal lobes dominate the auditory function, and the posterior central gyrus dominates the tactile function [50,51]. Therefore, it is preferable to perform channel selection on an individual basis using detection accuracy to optimize the channel subset. Moreover, an eight-channel subset has produced tantamount accuracies to the traditional 32-channel complete set [52].

**Figure 5:**
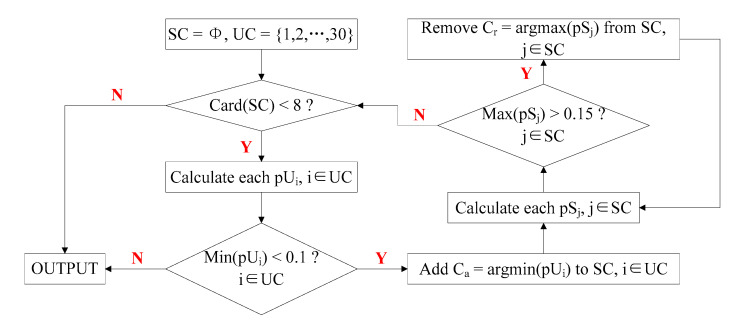
Flow diagram of Jumpwise regression algorithm. The value pU is the *p*-value of the partial F-test statistics of the model containing all features of the selected channel set (SC) against the features in SC except those belonging to the unselected channel set (UC), and the value pS is the *p*-value of the partial F-test statistics of the model containing all features of SC against the features in SC except those belonging to SC. The *p*-value thresholds for adding and removing channels were set to 0.1 and 0.15, respectively.

As shown in Figure 5, we adopted the Jumpwise regression algorithm to obtain optimal eight-channel subset [53]. Initially, the selected channel set (SC) and the unselected channel set (UC) are defined as an empty set and a complete set with 30 elements (30 in 32 channels, except REF and GND), respectively. Then, the channel set SC is checked to determine whether it contains eight elements; if so, the results are output; otherwise, channels are added or removed to SC until the target channel number (i.e., eight) is attained. Additional details are shown in Figure 5.

### 3.3. BLDA Classifier

In our approach, we applied Bayesian linear discriminant analysis (BLDA) algorithm [54,55,56] to train the feasible classifier. BLDA algorithm is derived as follows:

Initially, we assume that the linear regression target *y* is related to the feature vector *x*, and we define the linear discriminant function as:

(2) $$y=w^{T}x+n$$

where *w* is the weight vector and *n* denotes Gaussian noise. The likelihood function of *w* can be defined as:

(3) $$p\left(D|\beta ,w\right)={\left(\frac{\beta }{2\pi }\right)}^{\frac{N}{2}}exp\left(-\frac{\beta }{2}||X^{T}w-y||\right)$$

where *D* represents the dimension of the feature space, *β* is the inverse of the noise variance, *N* is sample size and *X* denotes the horizontal superposition matrix of the feature vectors. In Bayesian method, the prior probability distribution of *w* can be defined as:

(4) $$p\left(w|\alpha \right)={\left(\frac{\alpha }{2\pi }\right)}^{\frac{D}{2}}{\left(\frac{\varepsilon }{2\pi }\right)}^{\frac{1}{2}}exp\left(-\frac{1}{2}w^{T}I’\left(\alpha \right)w\right)$$

where *I’*(*α*) is (*D* + 1) dimensional diagonal matrix defined as:

(5) $$I’\left(\alpha \right)=\left[\begin{array}{cccc} \alpha & 0 & \cdots & 0\\ 0 & \alpha & \cdots & 0\\ \vdots & \vdots & \ddots & \vdots \\ 0 & 0 & \cdots & \varepsilon \end{array}\right]$$

Therefore, the mean value *m* and the variance *C* of the posterior probability distribution of *w* can be respectively defined as:

(6) $$\{\begin{array}{c} m=\beta {\left(\beta XX^{T}+I’\left(a\right)\right)}^{-1}Xy\\ C={\left(\beta XX^{T}+I’\left(a\right)\right)}^{-1} \end{array}$$

In conclusion, we obtain the estimated value of the mean value *μ* and variance *σ*^2^ of the linear regression target *y*:

(7) $$\{\begin{array}{c} \mu =m^{T}\hat{x}\\ \sigma^{2}=\frac{1}{\beta }+{\hat{x}}^{T}C\hat{x} \end{array}$$

### 3.4. ERP Detection

In the online signal processing procedure, the BLDA classifier was used to calculate the scores of ERP responses based on the following equation:

(8) $$\{\begin{array}{l} score_{rk}=W^{T}X_{rk}\\ score_{ck}=W^{T}X_{ck} \end{array}$$

where r,c∈{1,2,3,4,5,6} are the indexes of row and column stimulus codes, respectively, k∈{1,2,3,⋯,K} is the trial number, *K* is the total trial number for each row/column index selection and *W* is the weight column vector of the BLDA classifier with 200 elements (8 channels by 25 time points). Before calculating the ERP scores, each preprocessed data segment was reshaped into a column vector *X* with the same elements as *W*. In addition, the scores of each row/column code were obtained by averaging all the scores with the same code number.

(9) $$\{\begin{array}{l} Score_{r}=\frac{1}{K}\overset{K}{\underset{k=1}{\sum }}score_{rk}\\ Score_{c}=\frac{1}{K}\overset{K}{\underset{k=1}{\sum }}score_{ck} \end{array}$$

where *K* is the total trial number for the current selection of row/column index. Then, the row and column indexes were determined by the maximum scores of the ERP response, which are denoted as:

(10) $$\{\begin{array}{l} Index_{r}=\underset{r\in \left\{1,2,3,4,5,6\right\}}{argmax}\left(Score_{r}\right)\\ Index_{c}=\underset{c\in \left\{1,2,3,4,5,6\right\}}{argmax}\left(Score_{c}\right) \end{array}$$

Eventually, the target character was selected based on the intersection of the row and the column index in the given character matrix (see Figure 1a):

(11) $$TARGET=\left(Index_{r},\;Index_{c}\right)$$

### 3.5. Selection of Optimal Trials

ITR is the most common standard to evaluate the system performance in BCI literature [1]. It is defined as:

(12) $$\mathrm{ITR}=\left\{{log}_{2}N+A{log}_{2}A+\left(1-A\right){log}_{2}\frac{1-A}{N-1}\right\}/T$$

where *N* is the total number of all characters (i.e., 36), *A* represents the selection accuracy rate and *T* denotes the time interval (including 1 s for the break) per selection. In this study, the optimal trial number was selected by maximizing the ITR of each subject. To avoid overestimation, we adopted leave-one-block-out cross validation (LOOCV) to obtain the ITR for each subject. Specifically, we utilized nine runs for training and the last run for testing to calculate the ITR. To reduce fatigue and avoid extensive waiting periods, the maximum number of trials was set to ten. Then, the optimal number of trials per subject was determined as the number of repetitions which the ITR was at a local maximum. Finally, the optimal number of trials was used in the online signal processing.

### 3.6. Statistical Analysis

In this study, SPSS software (IBM SPSS statistics, IBM Corporation, Armonk, NY, USA) was used for statistical analysis. One-way repeated-measures analysis of variance (ANOVA) was applied to test the difference for classification accuracy, the number of trials and ITR between different methods (bimodal, auditory and tactile BCI), respectively. If the data did not conform to the sphericity assumption obtained by Mauchly’s test of sphericity, the Greenhouse–Geisser correction was performed. All post hoc pairwise comparisons were Bonferroni corrected. The alpha level was set at 0.05.

## 4. Results

### 4.1. Online Performance

In Table 1, the online performances of the ERP-BCIs using auditory, tactile and bimodal stimuli are presented. The experimental results demonstrated that the online performance of the bimodal approach was significantly better than that of each unimodal (i.e., auditory and tactile). One-way repeated measures ANOVA showed there was significant difference of ITR (F(2,33) = 5.2, *p* < 0.05) between three methods (bimodal, auditory and tactile). However, one-way repeated measures ANOVA showed there was no significant difference for the classification accuracy and the number of trials between three methods. Specifically, compared with the auditory (8.63 bit/min) and tactile approach (8.53 bit/min), the average ITR of the bimodal BCI reached 11.66 bit/min, an improvement of 35.11% and 36.69%, respectively. Moreover, the bimodal BCI resulted in a higher mean classification accuracy (72.78%) than that of each unimodal approach (auditory: 63.89%; tactile: 70.00%) with a fewer average number of trials (bimodal: 2.33; auditory: 2.58; tactile: 3.00). For S1 and S2, the ITR of the tactile BCI was superior to those produced by the auditory and bimodal BCIs, which suggest that tactile stimulus is preferable for these subjects.

### 4.2. Offline Analyses

**Figure 6:**
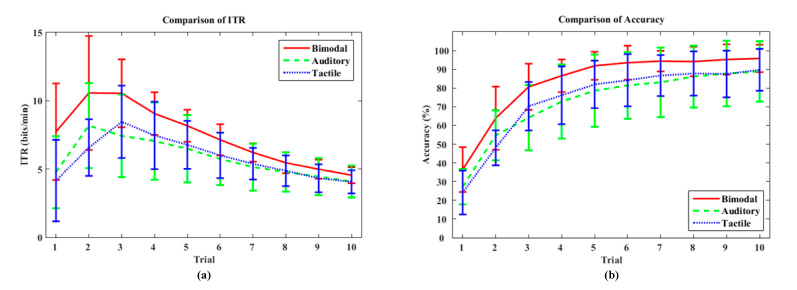
Offline ITR (**a**) and classification accuracy (**b**) of auditory, tactile and bimodal BCI using the first ten runs of each session. Error bars denote the standard deviation.

We performed the offline training performance comparisons among the auditory, tactile and bimodal in terms of ITR and classification accuracy. We applied a standard leave-one-block-out cross validation (LOOCV) to evaluate the offline performance. Specifically, there were 10 blocks (here, a block is a run: see Section 2.4) for each subject in the offline training data, and 10 estimations of the classifier performance were conducted. In each calculation, nine blocks were used for training, and the remaining one was tested. Therefore, the leave one block out cross validation here was equivalent to 10-fold cross validation. As shown in Figure 6, both the average ITR and the classification accuracy of the bimodal BCI were consistently higher than those of each unimodal BCI across all repetitions. One-way repeated measures ANOVA showed there was a significant difference for ITR (*p* < 0.05) and the classification accuracy (*p* < 0.05) in each repetition of trial between three methods (bimodal, auditory and tactile). In addition, with increasing trial repetition number (i.e., an increase in *T*), the classification accuracy increased rapidly before converging to an asymptote. Hence, we are able to select the optimal number of trials for each subject by selecting the point at which the ITR exhibits a local maximum by assessing the offline performance of each subject. These results indicate that the overall system performance of the bimodal BCI is superior to each unimodal BCI.

## 5. Discussions

### 5.1. Classification Accuracy of Each Direction

**Figure 7:**
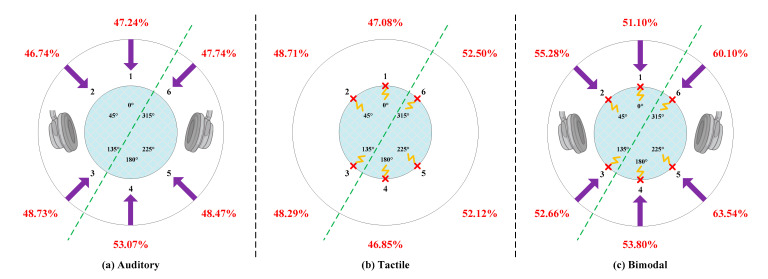
Classification accuracy comparisons among the six directions of auditory (**a**), tactile (**b**), and bimodal (**c**) approaches. The green line divided the stimulus area into the right-posterior part (“4”, “5” and “6”) and the left-anterior part (“1”, “2” and “3”) for the ease of analyzing significance.

The classification accuracy comparisons among the six directions of bimodal, auditory and tactile approaches are illustrated in Figure 7. To further discuss whether the classification performance differed across the six directions, we obtained the mean three-trial classification accuracy of each direction for all the subjects. A ten-fold cross-validation procedure was applied to evaluate the accuracy using all runs in each session. We selected the three-trial accuracy as a benchmark measure since all of the modalities achieved minimum accuracies required for effective communication (i.e., each was higher than 60%) when using three trials at least (See Figure 6b).

As shown in Figure 7, the classification accuracies of the bimodal BCI were higher than the corresponding accuracies of each unimodal BCI for all directions, which further validates the effectiveness and utility of the proposed bimodal BCI. In addition, although the same stimulus intensity (i.e., the playing volume and the current intensity) was delivered to the subjects in each direction, the mathematical statistical results indicate that the average accuracy of the right-posterior area (“4”, “5” and “6”) was significantly higher than that of the left-anterior area (“1”, “2” and “3”) for each modality (*p* < 0.05 for auditory; *p* < 0.05 for tactile; *p* < 0.01 for the bimodal) by using an one-way repeated measures ANOVA. We attempt to further explore the reasons for the superior performance observed using the bimodal BCI system as follows:

- In total, 80% of the information received by human beings is from the visual system and the rest is collectively referred to as non-visual information. Because our eyes are in the anterior area, we cannot focus on the posterior area in a visual way. As previous research indicates, auditory and tactile stimuli play a quite important role in enhancing human spatial perception of the posterior area, whereas auditory and tactile are not crucial for target identification within the range of human vision [57,58]. Hence, the posterior area was superior to the anterior area for non-visual ERP-BCIs, especially auditory and tactile ERP-BCIs.
- Tommasi and Marzoli demonstrated that the human brain processes sounds heard from our two ears differently. Signals received by the right ear are processed first and generally commands received by our right ear are easier to execute. This is defined as right-ear advantage [59] or multisensory right-side-of-space advantage [60]. To be specific, there might be a right-ear advantage in dichotic listening compared to the left in most individuals, which leads to the phenomenon that the left hemisphere of the brain is used for language processing and may be more sensitive to right-sided stimuli [61]. Consequently, the detection accuracy of right-sided ERP targets was superior to targets presentation on the left side.

### 5.2. Results of Channel Selection

**Figure 8:**
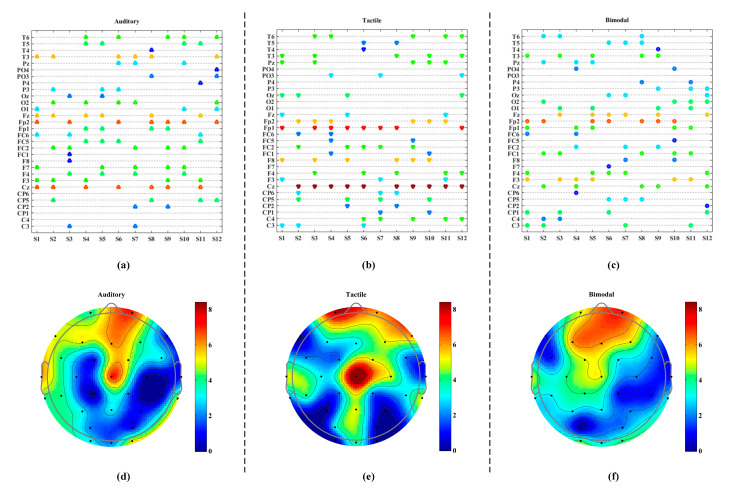
The results of channel selection for each subject. (**a**–**c**) denotes the eight optimal channels of each subject for the three modalities; (**d**–**f**) represents the total number of selected channels on the scalp for the twelve subjects. The color bar from dark blue to dark red indicates the summed number of channel selections for each electrode from zero to eight times, respectively.

We illustrated the eight optimal channel sets for each subject in the form of scatter diagrams and brain topographic maps. As shown in Figure 8a–c, the selected channels did vary from individual to individual across all the three stimulus modalities, thus validating the need to implement channel selection on a per subject basis.

In particular, the four most selected channels for the auditory, tactile and bimodal stimulus approaches were (Cz, Fp2, Fz and T3), (Cz, Fp1, Fp2 and F8) and (Fp2, Fz, F3 and T3), respectively. Fp2, Fz, Cz and T3 were the four most frequently selected channels among the three stimulus modalities. In Figure 8d–f, we found that Fp2 (located at right frontal pole) remained crucial for most subjects regardless of modality. Then, this finding corroborates the research on spatiotemporal analysis of ERPs by Cao et al., suggesting that the preparation effects appear in the right frontal pole first followed by the left pole [62]. We also noted that the Fz position (frontal midline) was often selected in the auditory and bimodal stimulus conditions. This selection preference to Fz could be explained through prior research, in which Gill et al. and Choi et al. determined the signals in the medial frontal lobe area can be applied to detect the surprisal response [63,64]. In addition, the channel Cz was selected for most of the subjects in the auditory and tactile ERP-BCIs (especially in the tactile case where it was chosen in 10 of 12 subjects). This observation is remarkably similar to previous findings that the tactile function area is primarily located in central area of the brain [34]. Another fact was that the channel T3 (left hemisphere) was selected more frequent than T4 (right hemisphere) for both the auditory and bimodal approaches. This finding is consistent with other previous studies [65,66,67,68], suggesting that the auditory functional areas are located in left temporal lobe areas in 95% of humans. Furthermore, the channels selected in the bimodal stimulus approach tended to consist of a union of channel used in the auditory and the tactile approaches. Hence, the performance improvement observed in the bimodal ERP-based BCI may be attributable to simultaneous activation of both the sensorimotor and the auditory cortices. In conclusion, the procedure of channel selection effectively reduced the computational complexity and the classification performance of the BCI was improved by means of combining the auditory and tactile stimuli.

### 5.3. Limitations and Optimized Orientations

Our major objective is to establish approaches to further improve the performance of an auditory-tactile bimodal ERP-BCI system. The limitations of current study and the corresponding optimized orientations are discussed as below:

- The current optimal number of trials is estimated using offline calibration data and is fixed prior to the online use. During online signal processing, the target is determined once the optimal number of trials is met. However, the brain state as well as individual electrode conditions change over time. The optimal number of trials determined offline may not be optimal for the subsequent online experiment. Therefore, the number of trials can be further optimized by applying dynamic stopping strategy, which adaptively determines a selection time in each single character selection [69].
- The performance of the bimodal BCI system proposed in this paper is superior to the auditory or tactile BCI systems alone. However, we still do not know the underlying basis for this superior performance. As previous researches indicated, Xu et al. optimized the performance of visual-based BCI systems by combing the P300 and SSVEP features [70]. Therefore, in our future work, we will attempt to extract more potentials, such as steady state somatosensory evoked potential (SSSEP) and steady state auditory evoked potential (SSAEP), to analyze the dynamic information of human brain neural activity under the condition of delivering the auditory-tactile bimodal stimuli.
- Recently, BCI technology has been widely used in neural system and rehabilitation engineering especially as a potential communication solution for persons with severe motor impairments. Traditional visual-based BCI systems are dependent on the user’s vision and it is thus difficult to apply these solutions to the patients with visual impairments. The proposed BCI approach provides an alternative way to establish a visual-saccade-independent online brain-computer cooperative control system based on multisensory information. While the proposed approach is currently still in the laboratory phase, it is hoped that the system can be easily adapted to real-world applications.

## 6. Conclusions

A novel 36-class auditory-tactile bimodal ERP-BCI system was proposed in this paper, in which six-virtual-direction audio files were delivered through the headphones and six-position electro-tactile stimuli were delivered from the corresponding directions simultaneously. Moreover, we used Jumpwise regression to select the optimum subset of eight channels, and trained a BLDA classifier for automated target detection. The optimal number of trials for each subject was also tailored and used in the online process. The mean online ITR of the bimodal stimulus BCI reached 11.66 bit/min, increases of 35.11% and 36.69% compared with the auditory (8.63 bit/min) and tactile (8.53 bit/min) BCIs, respectively. The results demonstrate the performance of bimodal system was superior to each unimodal system (i.e., auditory and tactile). As far as we know, the bimodal stimulus BCI has achieved the highest number of possible characters (i.e., 36-class) and the highest reported ITR (11.66 bit/min) for a non-visual ERP-BCI to date. We believe that the proposed paradigm should be feasible for the patients with severe motor impairments who are currently ill-served by existing gaze-dependent BCI systems.

## Figures and Tables

**Table 1:** Online performance comparisons (Acc. (%), information transfer rate (ITR) (bit/min)).

Subject	Auditory			Tactile			Bimodal		
	Trial	Acc.	ITR	Trial	Acc.	ITR	Trial	Acc.	ITR
S1	2	66.67	10.90	3	100.00	15.43	2	80.00	14.68
S2	2	73.33	12.72	3	100.00	15.43	2	80.00	14.68
S3	1	33.33	6.34	3	66.67	7.58	1	46.67	10.96
S4	2	53.33	7.63	3	73.33	8.85	2	80.00	14.68
S5	2	66.67	10.90	3	60.00	6.41	2	80.00	14.68
S6	2	60.00	9.21	3	66.67	7.58	2	66.67	10.90
S7	1	33.33	6.34	2	46.67	6.17	2	66.67	10.90
S8	4	73.33	6.78	4	73.33	6.78	3	73.33	8.85
S9	4	73.33	6.78	3	73.33	8.85	5	100.00	9.59
S10	5	86.67	7.27	3	53.33	5.31	3	80.00	10.21
S11	4	80.00	7.83	2	46.67	6.17	3	73.33	8.85
S12	2	66.67	10.90	4	80.00	7.83	1	46.67	10.96
Mean	2.58	63.89	8.63	3.00	70.00	8.53	2.33	72.78	11.66
Std	1.26	15.98	2.11	0.58	16.89	3.25	1.03	14.27	2.25
